# Editorial: Sensory Stimulation and Oxytocin: Their Roles in Social Interaction and Health Promotion

**DOI:** 10.3389/fpsyg.2022.929741

**Published:** 2022-06-15

**Authors:** Kerstin Uvnäs Moberg, Henri Julius, Linda Handlin, Maria Petersson

**Affiliations:** ^1^Department of Animal Environment and Health, Swedish University of Agricultural Sciences, Skara, Sweden; ^2^Department of Special Education and Rehabilitation, University of Rostock, Rostock, Germany; ^3^School of Health Sciences, University of Skövde, Skövde, Sweden; ^4^Endocrine and Diabetes Unit, Department of Molecular Medicine and Surgery, Karolinska Institutet, Stockholm, Sweden

**Keywords:** oxytocin, sensory nerves, sensory stimulation, social interaction, anti-stress effects, health, birth, lactation

## Introduction

The aim of this call was to collect papers describing how oxytocin may be released by different kinds of sensory stimulation to induce wellbeing and restorative processes and to inhibit pain, stress and inflammation. A large number of interesting articles of very high quality were received and 19 papers were accepted for publication.

All the included articles have contributed to expand the knowledge about oxytocin in a very substantial way both regarding its effect spectrum and regarding its association with sensory, somatosensory stimulation, in particular. In fact, the obtained data contribute to prove the hypothesis that the oxytocinergic system is a widespread integrative system, which is linked to social interaction, wellbeing, reduction of stress and pain as well as to reproductive, growth promoting and restorative effects. The activity of this archaic oxytocin system is under control of hormones and sensory nerves, which convey information regarding the state of the internal and the external environment. The oxytocin linked effects may be induced in the short-term as well as in the long-term perspective. All of the articles which were accepted and included in this issue, in their own unique way, contribute to describe oxytocin beyond its classical role in birth and milk ejection in accordance with the concept described above. We describe and discuss the data after having categorized the results presented in the articles according to certain subjects.

## The Structure of Oxytocinergic System

In order to facilitate the understanding of how oxytocin can exert such an important integrative function on physiological and behavioral function, a short summary of the distribution of the oxytocinergic system and its relationship to somatosensory innervation is presented.

Oxytocin is produced in large, magnocellular neurons of the supraoptic and the paraventricular nuclei (SON and PVN) of the hypothalamus and in small parvocellular neurons of the PVN. The magnocellular neurons project to the posterior pituitary from where oxytocin is released into the circulation, e.g., in connection with birth and lactation. The parvocellular neurons of the PVN consist of subgroups, one of which projects to the median eminence to influence pituitary hormones, such as ACTH and prolactin. The other subsets of parvocellular oxytocin neurons project to extrahypothalamic areas, such as the Locus Coeruleus (LC) the rostroventromedial medulla (RVLM), the preganglionic sympathetic neurons and to the dorsal vagal motor nucleus (DMX), the nucleus tractus solitarius (NTS), the periaqueductal gray (PAG), the dorsal column of the spinal cord as well as to parasympathetic ganglia in the lumbosacral region. Other oxytocin fibers project to the amygdala, the hippocampus, and some areas in the cortex (Sofroniew, 1983; Buijs et al., 1984; Sawchenko and Swanson, 1984). Oxytocin also reaches other areas in the brain *via* axon collaterals of magnocellular neurons from the SON and PVN (Stoop, 2012; Grinevich and Stoop, 2018). In addition, oxytocin is produced in and released from certain peripheral tissues and cells to exert local actions *via* paracrine mechanisms (Jankowski et al.). We refer to all of these sites of oxytocin production and their function to “the oxytocinergic system”.

### The Somatosensory Innervation of the Oxytocinergic System

Oxytocin release during birth (the Fergusson reflex) is mediated *via* parasympathetic afferents to the vagal sensory nucleus (NTS) and from there, *via* noradrenergic fibers (the A2 fibers) to the SON and PVN. Parasympathetic afferent nerves from the other parts of the genitourinary tract and as well from the gastrointestinal tract use the same pathway. Noradrenergic fibers project to the magnocellular and to separate divisions of the parvocellular neurons in the PVN, indicating that these oxytocin functions can be activated together or separately (Sofroniew, 1983; Buijs et al., 1984; Sawchenko and Swanson, 1984; Burbach et al., 2006; Uvnäs Moberg and Petersson, 2022; Takahashi).

Suckling/breastfeeding causes a release of oxytocin *via* activation of cutaneous sensory nerve fibers emanating in the nipple, which enter the spinal cord *via* the dorsal root ganglia (DRG). The pathway that mediates these effects to the SON and PVN is not entirely clear, but may involve the parabrachial nucleus and a beta-inhibin pathway (Burbach et al., 2006). In addition, a subpopulation of vagal afferents that originate in the skin on the breast and the chest are activated in response to suckling (Uvnäs Moberg and Petersson, 2022).

Stimulation of cutaneous afferents from most parts of the body give rise to oxytocin release and to an oxytocin linked effect spectrum (Uvnäs Moberg and Petersson, 2022). It is, however not entirely clear by which pathways the sensory nerves from the skin reach the SON and PVN. The cutaneous afferents enter the spinal cord *via* the dorsal root ganglia and then travel to the brain either in dorsal column or in the spinothalamic pathway. Toku Takahashi in his paper suggests that afferent stimulation of cutaneous nerves can increase oxytocin release and production from the SON and PVN *via* axon collaterals extending from nerves projecting to higher brain areas, such as the thalamus and the sensory cortex (Takahashi). These nerves may correspond to the unmyelinated C tactile (CT) fibers, which respond to stroking, but may also involve myelinated fibers (McGlone et al., 2012; Uvnäs Moberg and Petersson, 2022). It is also possible that information induced by tactile stimulation reaches the PVN and SON *via* the NTS, the vagal sensory nucleus, that receives the input from the entire parasympathetic nervous system (Takahashi). Gentle touch *via* activation of C tactile afferents is considered to be an important trigger of wellbeing in response to cutaneous stimulation, as such stimulation has been shown to activate areas within the anterior cingulate cortex, which is involved in positive emotions. A link between stimulation of these nerve fibers and oxytocin release has been assumed to occur, but the exact mechanism is not fully known (Walker et al., 2017).

## Several Types of Sensory Stimulation Stimulate Oxytocin Release

The pulsatile release of oxytocin in response to birth, breastfeeding/suckling and sex are the most well-known types of oxytocin release (Uvnas-Moberg et al., 2019; Uvnäs Moberg et al., 2020). In the present issue several others type of sensory stimulation were shown to give rise to oxytocin release. A release of oxytocin was demonstrated to occur during lactation in both mothers (Takahashi et al.; Wredle et al.) and in newborn lambs (Nowak et al.). In the offspring the suckling stimulus triggers oxytocin release by activating sensory nerves in the oral mucosa (Uvnas-Moberg et al., 1987). Oxytocin levels are also shown to increase after stimulation of afferent vagal nerves following ingestion of food (Wredle et al.) and *via* activation of cutaneous afferent nerves in response to foot massage (Chen et al.) and in response to acupuncture or transcutaneous electrical nerve stimulation (TENS) (Takahashi). Most likely activation of afferent sensory nerves from all parts in the body can trigger oxytocin release/function given that the right stimulus is applied.

### Birth and Lactation

Three papers describe the role of oxytocin in connection with birth and lactation. Toku Takahashi discusses how oxytocin released during birth induces maternal behavior (Takahashi). Yuki Takahashi and coworkers describe how the newborn's intensity of suckling controls the variability of maternal oxytocin release and thereby maternal milk production and weight gain of the newborn, which is a new and clinically important finding (Takahashi et al.). As shown by Nowak et al., a lamb's preference for its mother is dependent on suckling-induced oxytocin-release into the brain. Another conclusion that can be drawn from this data is that oxytocin is released both in the offspring and the mother in response to suckling. It is also at the same time released both into the peripheral circulation and into the brain to induce adaptive psychological effects.

### Oxytocin Release Is Influenced by Additional Sensory Stimulation

It is well known that stressful stimuli may counteract the progress of labor and the success of breastfeeding (Uvnas-Moberg et al., 2019; Uvnäs Moberg et al., 2020). In the present issue two articles demonstrate positive effects on oxytocin release during birth and lactation by concomitant sensory stimulation. One article in the present issue demonstrates how the progress of birth is enhanced by the presence of a supporting person or a doula, who provides the mothers with touch, warmth, empathy and support in connection with birth (Stjernholm et al.). An analogous potentiating effect by sensory stimulation on oxytocin release is demonstrated in the paper by Wredle et al. In this study the increase of the oxytocin release in response to the milking machine is enhanced if the cows are allowed to feed during milking, which induces vagal afference (Wredle et al.). It is possible that many types of concomitant sensory stimulation can facilitate oxytocin release in response to other types of sensory stimulation.

### Medical Interventions May Inhibit Oxytocin Release/Functions

It is also of clinical importance to note that, as mentioned by Toku Takahashi, medical interventions in connection with birth, such as Cesarean Section or epidural analgesia, may inhibit the release of oxytocin in connection with birth and thereby negatively impact oxytocin linked effects such as maternal interaction and bonding to the infant (Takahashi). In fact, as shown by Takahashi et al., even sustained effects may be seen. Rooting behavior which is performed by the baby in connection with breastfeeding 2 days after birth is reduced if the mother had received an epidural in connection with labor and epidural analgesia given together with an oxytocin infusion in connection with birth, significantly decreases the amount of oxytocin released by suckling 2 days later (Takahashi et al.).

## Oxytocin and Stress Reduction

It is well known that oxytocin inhibits stress by decreasing the function of the HPA axis *via* inhibition of CRF secretion in the PVN and by a decrease of ACTH secretion from the anterior pituitary. In addition, oxytocin inhibits the activity of the sympathetic nervous system (Uvnas-Moberg et al., 2014; Neumann and Landgraf, 2019). In this issue Toku Takahashi summarizes data showing that oxytocin inhibits the activity of the CRF-producing neurons in the PVN *via* activation of local GABAergic neurons which activate GABA A receptors on the CRF neurons (Takahashi).

In the present issue an oxytocin mediated reduction of ACTH and cortisol levels, as a reflection of decreased activity of the HPA axis, was observed both after feeding and milking in dairy cows in the study by Wredle et al. On the other hand, stroking of the abdomen in dairy cows, known to give rise to increased social interaction and decreased heart rate was not associated with increased circulating oxytocin levels. Nor was stroking linked to a decrease of ACTH levels, but still cortisol levels were lowered (Wredle et al.). These data suggest, that cortisol levels might have been regulated at a lower level than at the level of the hypothalamus/pituitary, perhaps *via* an oxytocin mediated inhibition of the sympathetic nervous system as discussed earlier (Uvnas Moberg et al., 2020). These results show that depending on the intensity and possibly on the origin of the sensory nerve stimulated, the entire oxytocin system or only parts of it, may be activated. Suckling may stimulate the entire oxytocin system including the peak shaped oxytocin release into the circulation emanating from the magnocellular neurons of the SON and PVN. There is a small subset of oxytocinergic neurons from the PVN, that project to the median eminence and from there oxytocin is delivered to the anterior pituitary (Sawchenko, 1985). In response to touch or stroking of the skin only the oxytocinergic fibers from the PVN, which project to extrahypothalamic areas may be activated. As described earlier in this editorial the oxytocin system is divided into different subdivisions, which may be activated together or independently of each other. Perhaps gentle touch of the skin is linked to this “lower and perhaps basic” oxytocin effect pattern. It Is also possible that the decreased cortisol levels induced by a supporting person during birth, as described in this issue, is mediated by this lower type of oxytocin effect pattern (Stjernholm et al.).

### Stress May Inhibit Oxytocin Release

In some situations, stress may inhibit oxytocin release. Carmassi et al., presented data showing that oxytocin levels are lower in patients with post-traumatic stress disorder (PTSD) than in controls. This effect is likely to be due to an inhibition of oxytocin release by high stress levels caused by trauma (Carmassi et al.).

## Restorative Effects of Oxytocin

A paper by Jankowski et al. reviews a multitude of oxytocin linked cardioprotective effects, such as reduction of the size of cardiac infarction and a facilitated functional recovery of heart function in response to administration of oxytocin. The beneficial effects involve among many others, positive metabolic effects, reduced inflammation, apoptosis and oxidative stress as well as protection of mitochondria (Jankowski et al.).

## Importance of Touch at a Young Age

Four papers in the present issue point to the importance of providing large amounts of tactile and other types of sensory stimuli to newborns and young children, as this may be linked to facilitated development and to lifelong amelioration of social competence, wellbeing and reduced stress levels and consequently to better mental and physical health in adulthood. Bigelow and Power demonstrated long-term positive effects on both mothers and their babies regarding social competence and stress reduction in response to extra skin to skin contact between mothers and their full-term babies during the first weeks of life. Some aspects of these effects, probably consequences of oxytocin release in response to skin-to-skin contact could be demonstrated even 9 years later.

A comprehensive review of the literature describing the immediate and also long-term effects of skin-to-skin contact or other types of closeness early in life is provided in the paper by Norholt. He also summarizes the knowledge concerning the mechanisms involved in these effects including the important role of oxytocin. In addition, the paper provides data supporting the importance of extended closeness between the baby and its parents in early life, e.g., by baby-carrying, for the development of future positive relationships, for secure attachment and possibly also for health (Norholt).

Carozza and Leong also review the important role of gentle or affectionate touch for the infant's psychosocial and neurophysiological development. The role of oxytocin is highlighted as is the role of the meso-corticolimbic dopamine and endogenous opioid systems, which aid the development of social cognitive processes (Carozza and Leong).

The paper by Devine et al. also points to the important role of touch and closeness in early life, by showing that individuals who have been under the care of social services during childhood, not only rated significantly higher levels of childhood trauma and lower levels of positive childhood touch, they also had a specific reduction in sensitivity to the affective value of C tactile fiber targeted touch. These results suggest that lack of nurturing touch in early life leads to blunted sensitivity to affective touch in adulthood (Devine et al.).

## Mental “Stimulation” of Oxytocin Release

In addition, to the somatosensory stimuli that trigger oxytocin release, a huge amount of data showing that mental stimuli can trigger oxytocin release are emerging.

Two papers in the present issue discuss the phenomenon of synchronization. Synchronization represents a specific complex type of interaction, which sometimes involves behavior, sometimes physiological variables (physiological synchronization or PS) and sometimes both, and which occurs in different types of relationships including that between a psychotherapist and a patient. In the paper by Palmieri et al., the possible role of oxytocin as a mediator of synchronization as well as the mechanisms and pathways involved are discussed. Papasteri et al. provided evidence of distinct mechanisms for behavioral vs. hormonal changes following social sensorimotor synchronization.

Oxytocin function may also be influenced by psychotherapy. Phyllis Klaus describes how a mother got traumatized in connection with birth, by being treated in an insensitive way by the staff and by being separated from her child. This trauma caused problems with her relationship with the child, indicating that oxytocin release and effects that normally occur in connection with birth had been hampered. She also describes how this trauma including the mother's problematic relationship with the child was resolved in a psychotherapeutic setting characterized by warmth and holding and thus by high levels of oxytocin (Klaus). There may be many more psychotherapeutic techniques that involve the healing aspects of oxytocin. This will be the subject of a new special issue in Frontiers in clinical psychology: Oxytocin: Its role in wellbeing, social interaction, bonding, stress and trauma.

## Interaction With Animals and Nature

Friendly interaction between humans and animals is linked to oxytocin release and oxytocin associated effects (Handlin et al., 2011; Beetz et al., 2012). In this issue interaction between elderly humans and dogs was demonstrated to be linked to increased finger temperature (Nilsson et al.). The increased finger temperature and as previously shown decreased heart rate and decreased blood pressure (Handlin et al., 2018), are likely to represent anti stress effects which may involve activation of the oxytocin system. Such effects may be induced by activation of touch receptors in the palms of the hands but also by mental sensory cues.

Two papers by Grahn and colleagues in this issue discuss how different types of nature have specific “archetypal properties”, that are attractive to humans and which influence human behavior and physiology (Ottosson and Grahn). The effect of nature to cause relaxation, antistress and restorative effects might well by due to an activation of certain aspects of the oxytocin system. This presumably archaic way of activating the oxytocin system may respond to certain features of nature that signal peace, calm, safety, richness etc. (Grahn et al.).

## Exogenous, Intranasal Treatment With Oxytocin Spray

In two of the papers the role of intranasal administration of oxytocin was studied. In the paper by Le et al., increased eye gaze and increased empathy was observed in response to intranasal administration of oxytocin. Looking at the eyes, and not at other parts of the face was linked to increased levels of empathy, perhaps suggesting that the increased levels of empathy are secondary to a release of endogenous oxytocin caused by eye gaze (Le et al.). In the second paper, Chen et al., demonstrated how intranasal oxytocin increased pleasantness of manual massage and neural responses in brain regions involved in reward, emotion and salience as well as in a number of sensory and motor processing regions.

Administration of oxytocin spray has been tried as a treatment for a variety of disorders, such as autism, schizophrenia and depression, sometimes giving rise to positive and sometimes to negative results (Domes et al., 2013). One problem with administration of oxytocin spray is that it is not fully known whether and how it enters the brain and which areas in the brain that are indeed reached by oxytocin given in response to such administration (Leng and Ludwig, 2016). Therefore, it might be more favorable for therapeutic purposes to activate the endogenous oxytocin system, e.g., by stimulation of cutaneous sensory nerves. When cutaneous afferent nerves are stimulated, oxytocin may be released. In this way oxytocin induced stimulation of social interaction, decreased levels of fear, stress, pain and inflammation and increased wellbeing as well as growth-promoting and restorative effects might be induced. Oxytocin release in response to specific types of cutaneous stimulation may therefore be a much more specific and efficient way of administering oxytocin than by giving intranasal oxytocin spray (Uvnäs Moberg and Petersson, 2022). Not only massage and touch, but also, as demonstrated in this issue, TENS (performed on hind legs) and acupuncture may in part act by activation of the oxytocin system *via* somatosensory nerves (Yoshimoto et al., 2012; Takahashi).

## Importance of Techniques for Adequate Measuring of Oxytocin Levels

It should be noted that the literature on oxytocin levels suffers from methodological problems regarding the specificity of the assays used. RIA is supposed to be the gold standard. Today, however, the less specific technique ELISA is often used. As this technique often gives rise to higher levels of oxytocin than does RIA and since the two techniques do not always detect changes in oxytocin levels in the same situations, results regarding oxytocin levels are sometimes confusing and difficult to interpret. When analyzing oxytocin levels, the method used for oxytocin determinations has to be considered. This problem also affects some of the studies included in this issue, in which the less specific measures of oxytocin levels obtained by ELISA are reported. For a more elaborate and comprehensive discussion of these analytical problems see Uvnas-Moberg et al., 2019; Uvnäs Moberg et al., 2020.

## Call for More Interdisciplinary Studies

As mentioned above some of the included studies are related to the classical oxytocin topics, i.e. birth and breastfeeding. The more “modern” oxytocin research involves several other effects of oxytocin and oxytocin release by other types of stimulation, e.g., cutaneous stimulation. A surprising observation is that researches who study the role of oxytocin during motherhood and those who study the more recent and broader aspects of oxytocin rarely refer to each other's publications, as if there was a barrier between these fields of research. This is unfortunate since, as described above, the sensory mechanisms involved in oxytocin release and the oxytocin effects are quite similar irrespective of situation and type of stimulation of oxytocin release. We recommend the researchers within different disciplines to cross these invisible barriers to take part of each other's studies. We also recommend researchers to read, and to include data from older studies, since unique and important data are often published in such articles.

## Author Contributions

All authors listed have made a substantial, direct, and intellectual contribution to the work and approved it for publication.

## Conflict of Interest

The authors declare that the research was conducted in the absence of any commercial or financial relationships that could be construed as a potential conflict of interest.

## Publisher's Note

All claims expressed in this article are solely those of the authors and do not necessarily represent those of their affiliated organizations, or those of the publisher, the editors and the reviewers. Any product that may be evaluated in this article, or claim that may be made by its manufacturer, is not guaranteed or endorsed by the publisher.
